# Dissection of the NKG2C NK cell response against *Puumala Orthohantavirus*

**DOI:** 10.1371/journal.pntd.0010006

**Published:** 2021-12-06

**Authors:** Hannes Vietzen, Svenja Hartenberger, Stephan W. Aberle, Elisabeth Puchhammer-Stöckl

**Affiliations:** Center for Virology, Medical University of Vienna, Vienna, Austria; NIAID Integrated Research Facility, UNITED STATES

## Abstract

**Background:**

Infections with the *Puumala orthohantavirus* (PUUV) in humans may cause hemorrhagic fever with renal syndrome (HFRS), known as nephropathia epidemica (NE), which is associated with acute renal failure in severe cases. In response to PUUV-infections, a subset of potent antiviral NKG2C^+^ NK cells expand, whose role in virus defence and pathogenesis of NE is unclear. NKG2C^+^ NK cell proliferation is mediated by binding of NKG2C/CD94 to HLA-E on infected cells. The proliferation and activation of NKG2C^+^ NK cells via the NKG2C/HLA-E axis is affected by different NKG2C (NKG2C^wt/del^) and HLA-E (HLA-E*0101/0103) alleles, which naturally occur in the human host. Homozygous (NKG2C^del/del^) and heterozygous (NKG2C^wt/del^) deletions of the NKG2C receptor results in an impaired NKG2C/CD94 mediated proliferation and activation of NKG2C^+^ cells. We therefore analyzed the PUUV-mediated NKG2C^+^ NK cell responses and the impact of different NKG2C and HLA-E alleles in NE patients.

**Methodology/Principal findings:**

NKG2C^+^ NK cell expansion and effector functions in PUUV-infected cells were investigated using flow cytometry and it was shown that PUUV-infected endothelial cells led to a NKG2C/CD94 mediated NKG2C^+^ NK cell activation and expansion, dependent on the HLA-G-mediated upregulation of HLA-E. Furthermore, the NKG2C^del^ and HLA-E*0101/0103 alleles were determined in 130 NE patients and 130 matched controls, and it was shown that in NE patients the NKG2C^wt/del^ allele was significantly overrepresented, compared to the NKG2C^wt/wt^ variant (p = 0.01). In addition, *in vitro* analysis revealed that NKG2C^wt/del^ NK cells exhibited on overall a lower proliferation (p = 0.002) and lower IFNγ expression (p = 0.004) than NKG2C^wt/wt^ NK cells.

**Conclusions/Significance:**

Our results corroborate the substantial impact of the NKG2C/HLA-E axis on PUUV-specific NK cell responses. A weak NKG2C^+^ NK cell response, as reflected by NKG2C^wt/del^ variant, may be associated with a higher risk for a severe hantavirus infections.

## Author summary

Infections with *Orthohantaviruses* are an emerging worldwide public health concern. Infections with the *Puumala Orthohantavirus* (PUUV) may cause hemorrhagic fever with renal syndrome (HFRS), termed nephropathia epidemica (NE), which is associated with acute renal failure in severe cases. The knowledge about potentially protective immune responses against PUUV in the human host is still limited. The present study contributes to the understanding of NK cell based human immune responses against PUUV. Based on *in vitro* NK cell proliferation and activation experiments as well as on analysis of genetic risk factors in NE patients, we provide evidence that the NKG2C^+^ NK cell mediated immune responses are an important factor in the prevention of severe PUUV-infections. These findings do not only contribute to the deeper understanding of the PUUV-specific NK cell responses, but also identify a potential protective immune mechanism, which may also contribute to strategies for PUUV vaccine development.

## Introduction

Infections with *Orthohantavirus* are an emerging worldwide public health concern, affecting approximately 200.000 people annually in the Americas, Asia and Europe. In severe cases, hantavirus infections may lead to hantavirus hemorrhagic fever with renal syndrome (HFRS) or hantavirus cardiopulmonary syndrome (HCPS) [1]. *Puumala orthohantavirus* (PUUV) is the most common hantavirus in Europe. It is transmitted by bank voles (*Myodes glareolus*) [2] and may cause flu-like symptoms and in 20–30% of the cases may lead to nephropathia epidemica (NE), characterized in severe cases with an acute renal failure [3]. In the human host, PUUV infects, amongst other cell types, endothelial cells [4, 5]. Although no direct cytotoxic effect can be observed *in vitro*, PUUV-infections of endothelial cells are associated with the induction of an antiviral state, which involves on one hand a strong intrinsic immune response and on the other hand the upregulation of classical and non-classical HLA-molecules [6, 7].

A recent study in patients with acute NE showed that PUUV infection may lead to the expansion of the NKG2C^+^ natural killer (NK) cells subset [8]. This was previously observed only in human cytomegalovirus (HCMV) infections, however, most of the study patients were HCMV-seropositive as well [8]. NKG2C^+^ NK cells are characterized by the absence of inhibitory NKG2A/CD94 and high expression levels of activating NKG2C/CD94 receptors [9]. The expansion of NKG2C^+^ NK cells is mediated by the interaction of NKG2C with its cellular ligand HLA-E and by complex formation with the leader sequence of MHC-1, such as HLA-G [10] or the HCMV- encoded UL40 peptide, respectively. To date, a viral ligand of HLA-E, which leads to the activation of NKG2C/CD94 has only been identified for HCMV [11]. NKG2C^+^ NK cells provide a potent antiviral phenotype, characterized by a high cytotoxic potential and the high-level secretion of pro-inflammatory cytokines, such as IFNγ and TNFα [12].

The presence of distinct HLA-E and NKG2C alleles may however influence the peptide presentation and NK cell responses in NE patients. Heterozygous or homozygous deletion of NKG2C decreases the expression of NKG2C, leading to an impaired expansion of NKG2C^+^ NK cells in HCMV-infected individuals [13]. HLA-E*0101 and HLA-E*0103 alleles display different expression levels and different binding affinities to peptides [10].

This study elucidates the functional basis of PUUV-specific NKG2C^+^ NK cell responses and the relationship between NKG2C and HLA-E alleles in NE patients.

## Materials and methods

### Ethics statement

The study was approved by the institutional review board of the Medical University of Vienna (EK No. 1389/2019). Written/oral consent was not obtained due to the retrospective study design. All data were analyzed anonymously.

### Study cohort

In this study, we included 130 patients with NE, as defined previously [14] and which were hospitalized in Austria between 2014 and 2018. PUUV-infection was confirmed by PCR [14] as well as PUUV-specific IgM- and IgG-antibodies (both: recomLine Bunyavirus IgG/IgM, Mikrogen). All patients had fever, myalgia, malaise, abdominal pain, polyuria and headache, as characteristic NE cases [14]. Also, 130 PUUV IgG-negative controls matched for age, sex, and HCMV serostatus were included (Table 1). NK cells were procured from 24 naïve hantavirus blood donors for *in vitro* testing.

**Table 1:** Characteristics of the Study Cohort.

	Puumala-Virus- Infected NE Patients (N = 130)	Controls (N = 130)	p-value^1^
Median Age	44.3 (18–79)	42.9 (18–80)	ns
Female (%)	N = 38 (29.2%)	N = 38 (29.2%)	ns
HCMV-IgG seropositive (%)	N = 56 (43.1%)	N = 56 (43.1%)	ns
Puumala-virus Status			
IgG^-^, IgM^+^, PCR^+^ (%)	N = 25 (19.2%)		
IgG^+^, IgM^+^, PCR^+^ (%)	N = 88 (67.7%)		
IgG^+^, IgM^+^, PCR^-^ (%)	N = 17 (13.1%)		

*Abbreviations*: **HCMV:** Human Cytomegalovirus, **NE:** Nephropathia epidemica, **ns:** not significant

^1^ Differences between groups were assessed with the Mann-Whitney or χ^2^-test.

### HCMV serology

HCMV-specific IgG were measured in the plasma of NE patients, controls, and blood donors by ELISA (Euroimmune).

### Genotyping

Genomic DNA was isolated from plasma of NE patients, matched controls and hantavirus naïve blood donors using NucliSens EasyMag extractor (BioMérieux). DNA was eluted in 50μl of nuclease-free H_2_O. HLA-E*0101/0103 genotypes were determined by a Taqman assay and NKG2C^wt/del^ variants were determined by touchdown PCR as described before [15, 16].

### Viral titration

Briefly, Vero E6 cells (ATCC) were maintained in E-MEM with EBSS (BioWhittaker), and 10% FBS and infected at 0.01 MOI with the PUUV strain Sotkamo. After 14 days of infection, cell-free virus was harvested and stored at -80°C.

Pooled HUVECs (Lonza) were cultured using the EGM-2 BulletKit (Lonza) according to the manufacturer’s instructions. Prior to infection, the cells were cultured without hydrocortisone. For viral titration, 1.5x10^4^ cells were seeded in 96-well plates and incubated at 37°C with 50μL of 1:10 serially diluted viral concentrations for three days. Subsequently, cells were fixed with 80% acetone for 10 min and stained with mouse anti-hantavirus mAb A1C5 (1:100, Progen) and AF488 conjugated goat anti-mouse mAb (1:400, Invitrogen). Cell nuclei were counterstained with DAPI and counted using LAS X software (Leica Microsystems). Viral titers were calculated as infected cells per μL virus containing supernatant.

### HLA-surface stabilization assay

2.5x10^5^ HUVEC cells were seeded in 24-well plates and infected with PUUV laboratory strain Sotkamo (European Virus Archive) and a MOI of 1.5 or mock infected with UV-inactivated virus. After incubation for indicated time-points, HUVEC were harvested and either stained for the HLA-E or HLA-G surface expression by flow-cytometry. HLA-E or HLA-G expression levels were defined in comparison with unstained HUVEC.

### HLA-G knockout

HLA-G knockout in HUVEC cells was performed using HLA-G CRISPR/Cas9 KO and HLA-G HDR Plasmid (Santa Cruz) according to the manufacturer’s instruction. Successful HLA-G knockout was confirmed by HLA-G surface expression by flow-cytometry and qPCR, using a commercially available assay (ID: qHsaCED0020668, Bio-Rad Laboratories).

### NK cell proliferation assays

PBMCs were isolated from the whole blood of naïve hantavirus donors (Austrian Red Cross) using Ficoll-Paque centrifugation (GE Healthcare). CD56^+^/CD3^-^ NK cells were isolated by magnetic labeling of undepleted PBMCs (NK cell isolation kit, Miltenyi Biotec) followed by MACS column filtration (Miltenyi Biotec). Subsequently, 5x10^6^ cells were stored in aliquots containing FBS (Gibco) and 5% DMSO at—80°C until further analyses. Cells were thawed and placed overnight in RPMI 1640 medium supplemented with 10% FBS, 10 ng/mL IL-12 (PeproTech), and 100 ng/mL IL-18 (Biozym Scientific). RMA-S/LFA3/HLA-E cells (provided by C. Romagnani, German Rheumatism Research Centre Berlin, Germany) were maintained as previously described [11].

HLA-E surface stabilization was performed as previously described [11]. 2x10^6^ RMA-S/LFA3/HLA-E cells were incubated with 300 μM of indicated peptides (peptides&elephants) in 1 mL serum-free Opti-MEM (Thermo Fisher) at 37°C with 5% CO_2_ for 16h. Peptide-pulsed RMA-S/LFA3/HLA-E cells and CFSE (Thermo Fisher) stained NK cells (E:T: 2:1) were co-cultivated with 100 ng/ml IL-18 and 10 ng/ml IL-12 for 20h, followed by cultivation in RPMI 1640 medium with or without 10 ng/mL IL-15 for seven days. New 2x10^6^ peptide-pulsed RMA-S/LFA3/HLA-E cells were added each three days of co-culture. After co-culture all cells were harvested and analyzed by flow-cytometry.

For NK cell proliferation assays, pooled HUVECs (Lonza) were cultured using the EGM-2 BulletKit (Lonza) according to the manufacturer’s instructions. Prior to infection, HUVECs were cultured without hydrocortisone. 2.5x10^5^ cells were seeded in 24-well plates and incubated at 37°C over-night in a humidified incubator. Cells were infected with a MOI of 1.5 or treated with UV-inactivated virus for 72h hours. The viral suspension was afterwards removed and CFSE (Thermo Fisher) stained NK cells (E:T: 2:1) were co-cultivated with 100 ng/ml IL-18 and 10 ng/ml IL-12 for 20h, followed by cultivation in RPMI 1640 medium with or without 10 ng/mL IL-15 for seven days. After three days, NK cells were transferred to new PUUV-infected cells. After co-culture NK cells were harvested and analyzed by flow-cytometry.

### NK cell cytotoxicity assays

For NK cell cytotoxicity assays, pooled HUVECs (Lonza) were cultured using the EGM-2 BulletKit (Lonza) without hydrocortisone. 2.5x10^5^ cells were seeded in 24-well plates and incubated at 37°C over-night in a humidified incubator. Cells were infected with a multiplicity of MOI of 1.5 or treated with UV-inactivated virus for 72h hours. The viral suspension was afterwards removed and NK cells were added in a 2:1 (E:T) ratio in RPMI 1640 medium. In addition, 5μL of mouse-anti human APC-H7-CD107a mAB (BD Biosciences) was added. After one hour of co-culture, GolgiStop (BD Biosciences) was added according to the manufacturer’s instructions. After additional 6h of co-culture, NK cells were harvested and analyzed by flow-cytometry.

### Flow cytometry

Cells were fixed and permeabilized for flow cytometry using the FIX & PERM Cell Permeabilization Kit (Thermo Fisher). The following mouse anti-human conjugated antibodies were used: APC-Cy7-CD16, PE-Cy7-CD57, PE-CD94, APC-HLA-G, APC-HLA-E (BioLegend), BV421-CD56, BV510-NKG2A, PE-TNF, PE-Cy7-IFNγ and AlexaFluor647-NKG2C (R&D Systems). The mouse anti-hantavirus mAB A1C5 was coupled with a secondary AlexaFlour488 antibody using a commercially available labeling kit (AlexaFluor488 Antibody Labeling Kit, Thermo Fisher). Live cells were identified using 7-AAD (BD Biosciences) or a LIVE/DEAD cell viability assay (Thermo Fisher). FACS analysis was performed using the FACSCanto2 platform, FACSDiva version 6.1.2, and Flowjo version 7.2.5 (BD Biosciences).

### Statistical analyses

The distribution of patients’ gender and genetic variants was compared using χ^2^ test. The patients’ ages were compared using the Mann-Whitney U test. The proportions of proliferating NKG2C^+^ and NKG2A^+^ NK cells were analyzed using a paired t-test. ANOVA and Dunn’s post-test were used to analyze HLA-G- and HLA-E-expressing HUVECs at multiple time points. Baseline NKG2C^+^ were compared with the NK cell proliferation of distinct cell subsets using the coefficient of determination and the nonparametric Spearman test. P-values < 0.05 were considered significant. Statistical analyses were performed using IBM SPSS Statistics v24.0.

## Results

### PUUV infection leads to NKG2C^+^ NK cell expansions

**Fig 1:**
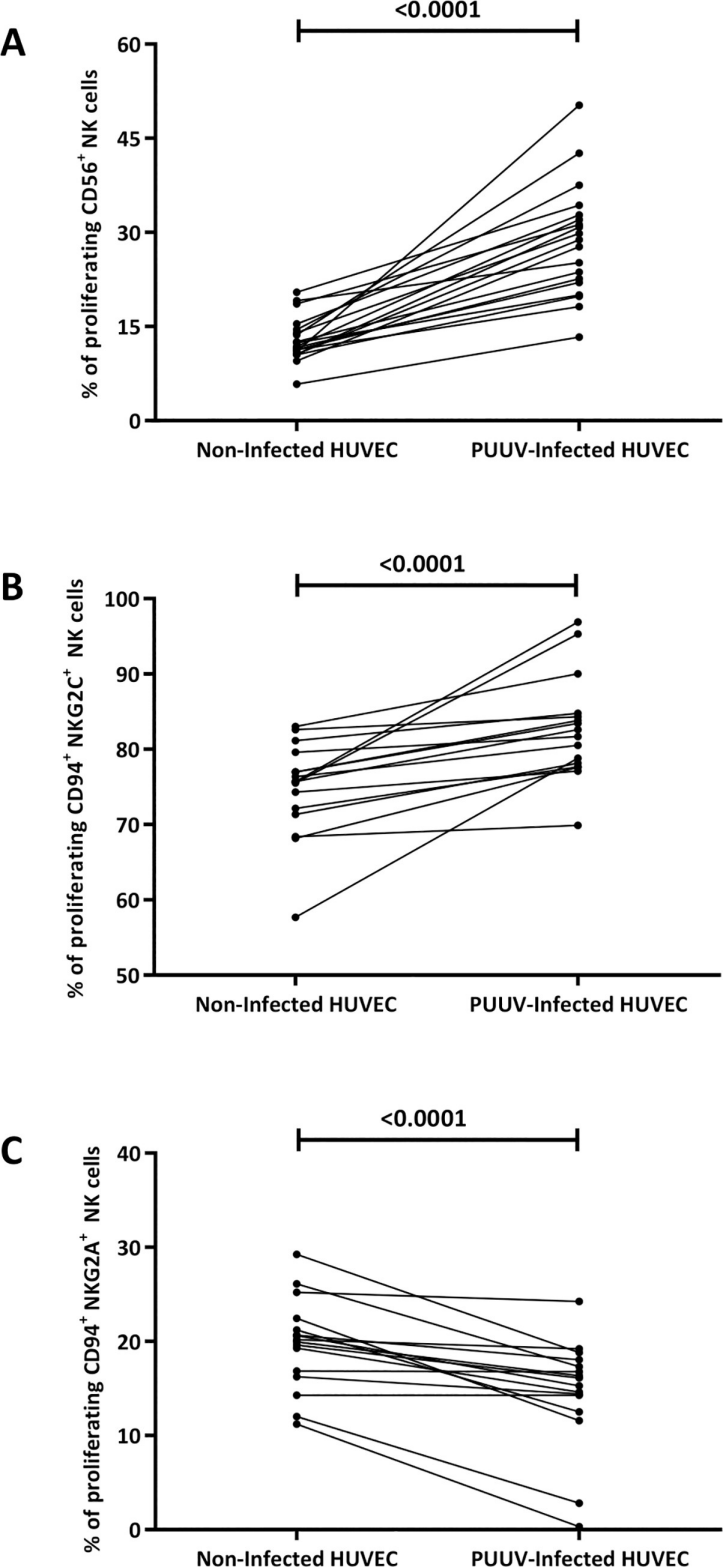
Comparison of the flow cytometry NK cell proliferation assays of NK cells derived from 18 healthy PUUV naive human donors when co-cultured with non-infected and PUUV-infected HUVECs in the presence of IL-15. Proliferation of (A) CD56^+^, (B) NKG2C+, and (C) NKG2A^+^ NK cells. Symbols represent individual donors. The percentage of proliferating NK cells in the presence of non-infected and PUUV-infected HUVECs was compared using paired t-test. **HUVEC**: human umbilical vein endothelial cells, **PUUV**: *Puumala orthohantavirus*.

To determine whether PUUV-infections leads to NKG2C^+^ NK cell expansion, we performed an *in vitro* stimulation of CFSE labeled CD56^+^ NK cells with PUUV-infected and non-infected human umbilical vein endothelial cells (HUVECs). In response to PUUV-infected cells and IL-15, an expansion of CD56^+^ (Figs 1A and S1) and NKG2C^+^ NK cells (Figs 1B and S1) but a contraction of NKG2A^+^ NK cells (Figs 1C and S1) was observed by flow-cytometry after seven days of co-culture. We have then assessed the phenotype of the proliferating NKG2C^+^ NK cells by analyzing the expression of the Fcγ-receptor CD16a, the differentiation marker CD57 and CD94 by flow-cytometry. The data are presented in S2A–S2C Fig. The proliferating NKG2C^+^ cells showed a significantly increased expression of FcγR3a/CD16a, a similar expression of CD57, and a lower expression of CD94 compared to those of proliferating NKG2A^+^ NK cells. Thus, the proliferating NKG2C^+^ NK cells were defined as CD56^+^CD57^+^CD94^low^NKG2C^+^NKG2A^-^CD16a^+^ NK cell phenotype. As a recently published study demonstrated that the CD56^+^ NK cell-mediated lysis of hantavirus-infected endothelial cells is highly dependent on IL-15 [17], we also tested the NKG2C^+^ NK cell proliferation in response to PUUV-infected cells, but in the absence of IL-15. As shown in S3A–S3C Fig, NKG2C^+^, NKG2A^+^ and total CD56+ NK cell expansion was significantly lower in the absence of IL15 and thus highly dependent on the presence of IL-15.

To further confirm that the expansion of NKG2C^+^, and to a lower extent also of NKG2A^+^ NK cells, is mediated via the NKG2C/CD94 or NKG2A/CD94 receptor activation, we stimulated CD56^+^ NK cells with PUUV-infected cells in the presence or absence of NKG2C- and NKG2A-specific monoclonal blocking antibodies. As shown in S3D Fig, neither NKG2C^+^ nor NKG2A^+^ NK cell proliferation was observed in the presence of respective blocking antibodies.

NKG2C^+^ NK cell expansion has only been observed in HCMV-infections or PUUV-infections in mostly HCMV-seropositive patients [8]. Therefore, we analyzed the HCMV serostatus of NK cell donors to determine its role in NKG2C^+^ NK cell expansion and assessed the NK cell expansion in response to PUUV-infected cells in association with the pre-existing HCMV serostatus of NK cell donors. Overall, no significant difference was observed between HCMV-seropositive and HCMV-seronegative donors in regard of total CD56^+^ NK cells, expansion of NKG2C^+^ NK cells and contraction of NKG2A^+^ NK cells. Especially the expansion of NKG2C^+^ NK cells and contraction of NKG2A^+^ NK cells showed, however, a notably greater range of proliferating NK cell subsets, derived from HCMV-seropositive compared to HCMV-seronegative donors (S4 Fig).

We further correlated the baseline NKG2C^+^ cell levels of all NK cell donors, HCMV seropositive and HCMV seronegative, with the increase of respective NKG2C^+^ NK cell subsets in response to PUUV-infected HUVEC. The baseline NKG2C^+^ cell levels were in overall: mean 5.3% (0.5% - 15.1%), for HCMV-seropositive donors: mean: 8% (1% - 15–1%), and for HCMV-seronegative donors: mean 2% (0.5%– 3.2%). The correlation between the pre-existing NKG2C^+^ NK cell subset and the proliferation of NKG2C^+^ in response to PUUV-infected cells was for all NK cell donors: r^2^ = 0.31 p = 0.08, for HCMV-seropositive NK cell donors: r^2^ = 0.29 p = 0.24, and for HCMV seronegative NK cell donors: r^2^ = 0.19 p = 0.59. Thus the pre-existing HCMV-serostatus had only a limited effect on the *in vitro* NKG2C^+^ NK cell proliferation in response to PUUV-infected cells.

### PUUV-specific NKG2C^+^ NK cell expansion requires HLA-G-dependent upregulation of HLA-E

**Fig 2:**
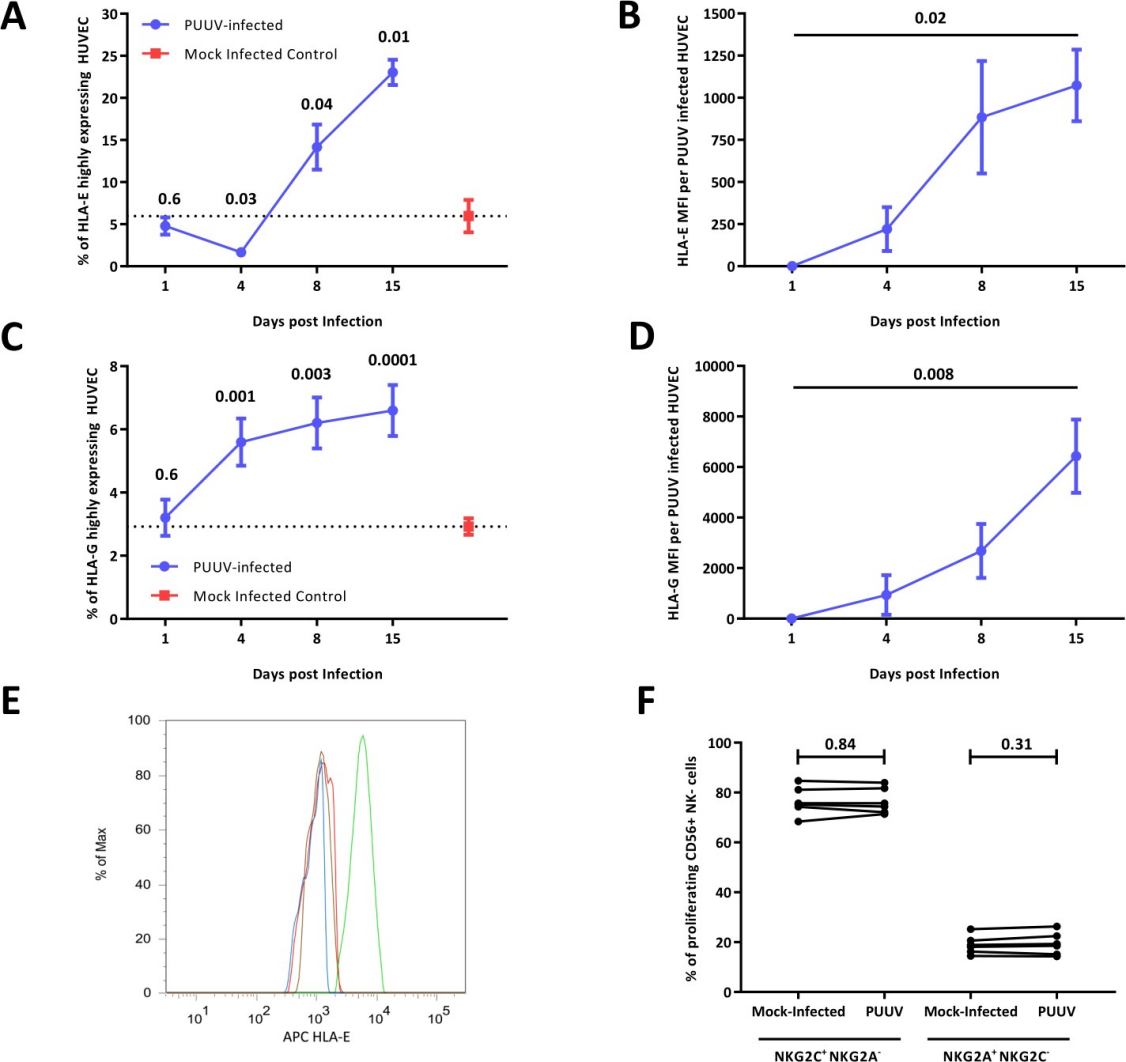
Expression of HLA-E and HLA-G in mock-infected HUVECs and PUUV-infected HUVECs measured using flow cytometry. (A) Percentage of HLA-E expressing HUVECs, evaluated after 1, 4, 8, and 15 days of PUUV or mock infection. (B) Expression levels of HLA-E per PUUV-infected cell evaluated after 1, 4, 8, and 15 days of PUUV infection. (C) Percentage of HLA-G expressing HUVECs evaluated after 1, 4, 8, and 15 days of PUUV or mock infection. (D) Expression levels of HLA-G per PUUV-infected cell evaluated after 1, 4, 8, and 15 days of PUUV infection. (A-D) Results are shown as means of three independent technical replicates ± SD. (A, C) ANOVA and Dunn’s post-test were used for statistical comparison between infected cells and mock-infected controls at single time points. (B, D) ANOVA was used for statistical comparisons of expression levels at all time points. (E) Representative example of three independent technical replicates showing HLA-E expression in PUUV-infected HUVECs (green), PUUV-infected HUVECΔHLA-G (blue), mock-infected HUVECΔHLA-G (red), and mock-infected HUVECs (brown). (F) Comparison of proliferation of NKG2C^+^ and NKG2A^+^ NK cells of six healthy NK cell donors after co-culturing with mock- or PUUV-infected HUVECΔHLA-G, respectively, in the presence of IL-15. Symbols represent individual donors. Paired t-test was used to compare the percentage of NK cells between mock-infected and PUUV-infected HUVECΔHLA-G, assessed using a single experiment per donor. **MFI:** mean fluorescence intensity, **PUUV**: *Puumala orthohantavirus*.

The peptide-dependent stabilization of HLA-E is essential for NKG2C/CD94-mediated NKG2C^+^ NK cell proliferation [8]. Therefore, we infected HUVEC cells with PUUV (S5 Fig) and analyzed the kinetics as well as the stabilization of HLA-E expression in response to PUUV infection. As observed using flow cytometry, the expression of HLA-E in live HUVEC increased over time, especially in PUUV-infected HUVECs (Figs 2A and 2B and S6).

In HCMV infections, NKG2C^+^ NK cell expansion is dependent on the presentation of a virus-derived UL40 peptide by HLA-E [11]. Therefore, we investigated the role of PUUV-encoded peptides presented via HLA-E in PUUV-associated NKG2C^+^ NK cell proliferation. We screened the PUUV-encoded peptides homologous to HCMV-UL40 peptides (YP_004928150.1, NP_941984.1, and NP_941983.1) and selected the most similar peptide (SRKPRNFLL). We also predicted the binding affinity of PUUV-encoded peptides to HLA-E using NetMHCpan and selected three candidates with the highest binding affinity to HLA-E (AIPPAAYSL, VLPDSLFSF, and LLPDKNLRM). We then performed an NK cell proliferation assay using RMA-S/HLA-E/LFA-E cells to study the impact of the four selected peptides on NK cell proliferation. However, NK cell proliferation was not observed (S7 Fig). Thus, the upregulation of an MHC-1 derived leader peptide may influence HLA-E stabilization in PUUV-infected HUVECs.

As an HLA-G-derived leader peptide can enhance NKG2C^+^ NK cell expansion [18], we studied the expression of HLA-G in live PUUV-infected HUVECs. PUUV infection increased the HLA-G expression levels in HUVECs (Fig 2C and 2D). To further assess the role of HLA-G-mediated upregulation of HLA-E in NKG2C^+^ NK cell expansion, we performed an HLA-G-specific knockout in HUVECs and reanalyzed the HLA-E expression and NK cell expansion in response to HLA-G-deficient PUUV-infected cells. HLA-G deficiency decreased the expression of HLA-E on the surface of PUUV-infected cells (Fig 2E) and inhibited NKG2C^+^ NK cell expansion (Fig 2F). Thus, HLA-G upregulation during PUUV infections is sufficient to induce NKG2C^+^ NK cell proliferation and effector functions.

### The NKG2C^wt/del^ variant is overexpressed in NE patients, leading to a severe PUUV infection

**Fig 3:**
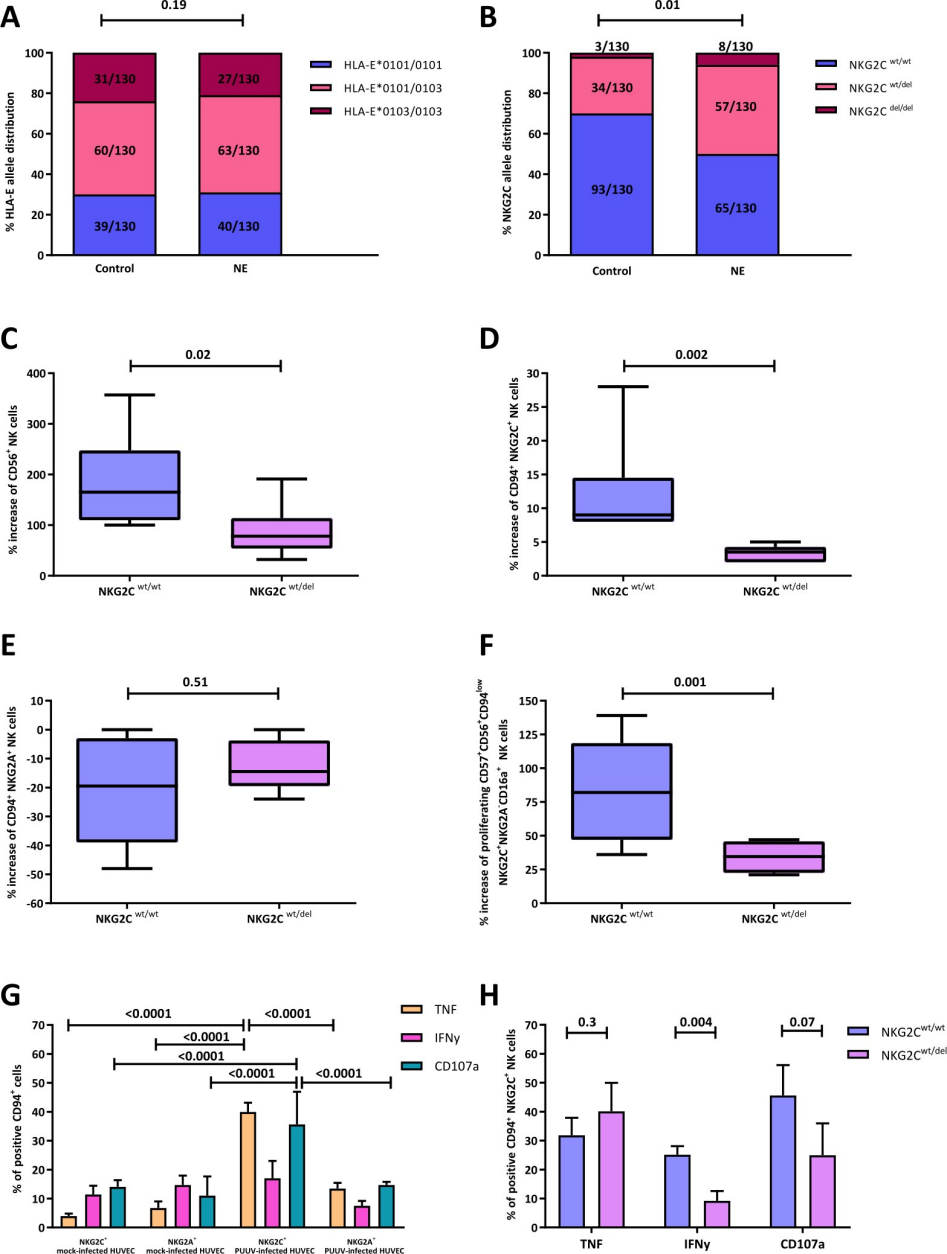
Comparison of the distributions of HLA-E (A) or NKG2C (B) genetic alleles in 130 NE patients and 130 matched healthy controls. Bars represent the relative frequency of (A) HLA-E*0101/0101, HLA-E*0101/0103, and HLA-E*0103/0103 alleles and (B) NKG2C^wt/wt^, NKG2C^wt/del^, and NKG2C^del/del^ alleles for each group. (A-B) χ^2^ test was used for statistical comparison of the alleles. (C-F) Comparison of NK cell proliferation between patients with NKG2C^wt/wt^ (N = 9) and NKG2C^wt/del^ (N = 9) statuses in the presence of IL-15. Comparison includes analyses of (C) CD56^+^ cells, (D) NKG2C^+^ cells, (E) NKG2A^+^ cells, and (F) CD57^+^CD56^+^ CD94^low^NKG2C^+^NKG2C^-^CD16a^+^ cells. Data of proliferating NK cells, expressing either the NKG2C^wt/wt^ (N = 9) or NKG2C^wt/del^ variant, are shown as median ± min/max. (G) Comparison of the expression levels of TNF, IFNγ, and CD107a of NKG2C^+^ and NKG2A^+^ NK cells of 12 healthy human donors (6 HCMV seropositive, 6 HCMV seronegative), either stimulated with mock- or PUUV-infected cells in the absence of IL-15. Data are shown as means of the 12 biological replicates ± SD. ANOVA and Dunn’s post-test were used for statistical comparisons of single activation markers between the subsets. (H) Expression levels of TNF, IFNγ, and CD107a of NKG2C^+^ NK cells stimulated with PUUV-infected cells in the absence of IL-15, and comparison of the results obtained from CD56^+^NK cells of six NKG2C^wt/wt^ (3 HCMV seropositive, 3 HCMV seronegative) and six NKG2C^wt/del^ (3 HCMV seropositive, 3 HCMV seronegative) expressing donors. Data are shown as means of six biological replicates ± SD. Mann-Whitney U test was used for statistical comparison. **Del**: deletion, **HUVEC**: human umbilical vein endothelial cells**, NE**: Nephropathia epidemica, **PUUV**: *Puumala orthohantavirus*, **wt**: wild type.

We then investigated the distribution of HLA-E*0101/0103 and NKG2C^wt/del^ genotypes in NE patients and controls. Although the distribution of HLA-E*0101/0103 genotypes was not significantly different (Fig 3A), NE patients carried the heterozygous NKG2C deletion variant more frequently than controls (p = 0.003, F-test, Fig 3B). Therefore, we investigated the role of NKG2C deletion in impaired NK cell expansion. We isolated NK cells from PUUV-negative donors, determined their NKG2C^wt/del^ status, and stimulated them with PUUV- or mock-infected HUVECs. Significantly higher amounts of CD56^+^ NK cells, NKG2C^+^ NK cells, and CD56^+^CD57^+^CD94^low^ NKG2C^+^NKG2A^-^CD16a^+^ NK cells were observed in NKG2C^wt/wt^ donors than in NKG2C^wt/del^ donors (Fig 3C–3F).

We then analyzed whether the effector response of NK cells depends on the NKG2C^wt/del^ status. NKG2C^+^ NK cells demonstrated in overall a significantly increased cytotoxicity in response to PUUV-infected cells, which was indicated by higher levels of CD107a and TNFα than those in NKG2A^+^ NK cells (Fig 3G). We compared the activation of NKG2C^wt/wt^ and NKG2C^wt/del^ NK cells in response to PUUV-infected HUVECs. NKG2C^wt/wt^ NK cells showed higher levels of IFNγ as compared to those in NKG2C^wt/del^ NK cells (Fig 3H).

## Discussion

In our study, we show that NKG2C^+^ NK cells expand in response to PUUV-infected cells and that those cells may play an important role in the defence against PUUV-infections in the human host.

So far, the expansion of NKG2C^+^ NK cells was only observed during HCMV-infection or during PUUV-infections in mainly HCMV-seropositive patients [8, 11]. We now demonstrate that NKG2C^+^ NK cell proliferation can also be induced by the cellular stress response in PUUV-infected cells. These findings are of special interest, as NKG2C^+^ NK cells provide a highly potent antiviral phenotype, which can lyse virally infected cells by direct or antibody mediated cytotoxicity and are furthermore an important source of pro-inflammatory cytokines, such as IFNγ or TNFα [9].

So far, distinct PUUV-specific immune responses, such as the PUUV-specific CD8+ T-cell response, were linked to the pathogenesis of PUUV-infection and some authors speculated that also NKG2C^+^ NK cells may contribute to NE disease severity [8]. However, our data show in contrast that NKG2C^+^ NK cells mediate protection from severe disease to a certain extent. In our NK cell activation experiments, only NKG2C^+^ NK cells responded against PUUV-infected cells via high a cytotoxicity response. In contrast, NKG2A^+^ NK cells showed in overall no specific activation towards PUUV-infected cells. Interestingly, PUUV developed several immune evasive strategies to evade virus specific immune responses and recently published *in vitro* data demonstrated a certain resistance of PUUV-infected cells against cytotoxic NK cell- mediated apoptosis [19, 20]. Whether the increased expression of cytotoxicity makers in NKG2C^+^ NK cells reflects also a better lysis of PUUV-infected cells *in vivo* requires further investigations.

As an additional major finding, we showed that the NKG2C/HLA-E axis has a substantial impact on the expansion of NKG2C^+^ NK cells, as blocking of the NKG2C receptor was associated with an absent proliferation of NKG2C^+^ cells in response to PUUV-infected cells. Thus, NKG2C/CD94 receptor stimulation is essential for NKG2C^+^ NK cell proliferation in response to PUUV-infected HUVEC. Interestingly, a recently published *in vitro* study demonstrated that the CD56^+^ NK cell mediated lysis of Hantavirus infected endothelial cells is also dependent on the expression and secretion of the pro-inflammatory cytokine IL-15 [17]. Taken together, these and our result demonstrate, that different stimuli ay activate distinct subsets of NK cells in hantavirus infected individuals. It remain thus unknown, which NK cell subsets may mediate protection from severe NE.

In our study cohort, natural deletion of NKG2C in the patients was furthermore associated with a higher risk for the development of NE. The overall distribution of homozygous or heterozygous NKG2C deletion was comparable to that of other European study cohorts [15, 21]. Studies investigating the role of NKG2C deletion in the defence against virus infections focused so far on HCMV, especially in solid organ and haematopoietic stem cell transplant patients and COVID-19 patients [15, 22, 23]. Our study further indicates that NKG2C deletion may play a role during severe PUUV-infections. We demonstrated that NKG2C^+^ NK cells, derived from healthy blood donors with the NKG2C^wt/wt^ variant showed a higher level of proliferation towards PUUV-infected cells. On a functional level, NK cells encoding for the NKG2C^wt/del^ variant showed lower levels of the IFNγ in response to PUUV-infected cells. Together these data raise the question, whether a strong NK cell derived IFNγ response is the main protective mechanism for severe PUUV-infection. A recently published study in mild and severe NE patients demonstrated that high IFNγ serum levels are associated with a mild form of NE [24]. The cellular effects of IFNγ are diverse and include the upregulation of MHC1 and MHC2 molecules, the induction of an antiviral state and the development of TH1 responses [25]. Interestingly, a recent study demonstrated that HUVEC cells infected with the related Hantaan Orthohantavirus (HTNV) highly upregulate classical MHC-1 molecules via expression of interferon type I [26]. Also, previously published studies demonstrated that hantavirus infections of endothelial cells elicit a strong intrinsic immunity [27]. It is a limitation of our study and remains to be addressed by future studies to evaluate to which extent the overall intrinsic immune responses as well as the interferon signaling of PUUV-infected cells contribute to the HLA-G mediated upregulation of HLA-E and the subsequent expansion of NKG2C^+^ NK cells in PUUV-infected individuals.

As an additional major finding, we demonstrate that the upregulation of HLA-E is associated with the upregulation of HLA-G, induced by PUUV-infection. So far, the expansion of NKG2C^+^ NK cells was observed during HCMV-infections, where the UL40 peptide shows some similarity to the MHC-1 derived leader peptides [11]. The HLA-G leader peptide VMAPRTLFL is known as strong activator of the NKG2C/CD94 receptor, but only as an intermediate activator of the inhibitory NKG2A/CD94 [28], which is consistent with our experimental data, which demonstrated the expansion of mainly NKG2C^+^ cells and the contraction of NKG2A^+^ NK cells.

Thus it appears that the upregulation of HLA-G during the cellular stress response alone is sufficient to induce NKG2C^+^ NK cell proliferation and effector functions. So far, NKG2C^+^ NK cells were regarded as a result of the imprint of HCMV on the human immune system. However, our data indicate that NKG2C^+^ NK cell proliferation and effector functions can also be induced independent of prior HCMV-infections. In contrast to the persistent infection with HCMV, from which sporadic reactivations and re-infections of HCMV are possible, PUUV-infections are cleared by the human immune system. It is unclear how long those cells are detectable in the human host without the recurrent stimuli for NKG2C^+^ NK cell proliferation, especially in HCMV-seronegative individuals. Overall, further studies are needed to evaluate to which extent a prior HCMV-infection increase the expansion of NKG2C^+^ NK cells in PUUV-infected individuals.

Interestingly, HLA-E is not only present on PUUV-infected endothelial cells, but can also be released as soluble HLA-E (sHLA-E) from the same cells. A recently published study found that endothelial cells induce the release of sHLA-E in response to TNFα, IFN-β and IFNγ [29], all which are released by PUUV-infected endothelial cells alone or PUUV-specific NK cell responses. It remain however unknown whether sHLA-E levels are influenced by PUUV-infections and which functional consequences altered sHLA-E levels on the PUUV-specific NK cell responses may have. Thus further studies are needed to evaluate the role of the sHLA-E during PUUV-infections.

In conclusion, the data provide evidence that the NKG2C/HLA-E axis has a substantial impact on the PUUV-specific NK cell response via the cellular stress response. Furthermore, it appears that an impaired NKG2C response, as described for NKG2C^wt/del^ patients, may favour the development of severe PUUV-infections.

## Supporting information

S1 Fig Gating strategy and determination of life (7-AAD^low^) and proliferating (CFSE^low^) CD56^+^ or CD94^+^ NKG2C^+^ or CD94^+^ NKG2A^+^ NK cells measured in response to PUUV-infected HUVEC. One representative example of 18 independent replicates is shown.
(TIFF)

S2 Fig Expression level of CD16, CD57, and CD94 in proliferating NKG2C^+^ and NKG2A^+^ NK cells measured in response to PUUV-infected HUVEC. Expression level of (A) CD16, (B) CD57, and (C) CD94 measured in proliferating NKG2C^+^ and NKG2A^+^ NK cells obtained from 18 different healthy PUUV-naive human donors in response to PUUV-infected HUVEC in the presence of IL-15. The data of the 18 samples are shown as median ± min/max. Paired T-Test was used for statistical comparison. **MFI:** mean fluorescence intensity
(TIFF)

S3 Fig Proliferation of CD56^+^, NKG2C^+^ or NKG2A^+^ NK cells in response to NK cells in the presence or absence of IL-15, α-NKG2C or α-NKG2A blocking antibody. (A-C) Proliferation of (A) NKG2C^+^ cells, (B) NKG2A^+^ cells or (C) CD56+ NK cells obtained from 18 different healthy PUUV-naive human donors measured in response to NK cells cultured either without (-PUUV-infected HUVEC) or with PUUV-infected HUVEC (+PUUV-infected HUVEC) in the presence or absence of IL-15. The median ± min/max of the results of the 18 samples is shown. ANOVA and Dunn’s post test were used to compare the percentage of NK cells between mock-infected and PUUV-infected HUVEC. (D) Proliferation of NKG2C^+^ and NKG2A^+^ NK cells obtained from 6 different healthy PUUV-naive human donors (3 HCMV seropositive, 3 HCMV seronegative) measured in response to NK cells in the presence of IL-15 and the presence or absence of either a α-NKG2C or α-NKG2A blocking antibody. The median ± min/max of the results of the 6 samples is shown. Paired T-Test was used to compare the percentage of proliferating NK cells between the presence or absence of respective blocking antibodies. **mAB**: monoclonal antibody, **PUUV**: Puumala-Virus.
(TIFF)

S4 Fig Comparison of NK cell proliferation of CD56^+,^ NKG2C^+^ and NKG2A^+^ NK cells between HCMV-seropositive and HCMV-seronegative donors. NK cell proliferation was measured in samples obtained from 18 different healthy PUUV-naive human donors, including HCMV-seropositive (N = 10) and HCMV-seronegative (N = 8) donors. Comparison of NK cell proliferation of (A) CD56^+^ (B) NKG2C^+^ and (C) NKG2A^+^ NK cells between HCMV-seropositive (N = 10) and HCMV-seronegative (N = 8) donors in the presence of IL-15. Data are shown as median of 18 different donors ± min/max. Mann-Whitney t-test was used to for statistical comparison between HCMV-seropositive and HCMV-seronegative donors. **HCMV:** Human Cytomegalovirus.
(TIFF)

S5 Fig Gating strategy and determination of viable (7-AAD^low^), PUUV-infected (Alexa488^+^) and HLA-G positive HUVEC obtained after 3 days post infection. One representative example of 3 independent replicates is shown.
(TIFF)

S6 Fig Kinetics of the PUUV-infection of HUVEC measured using flow cytometry. Kinetics of the PUUV-infection of HUVEC measured using flow cytometry. Percentage of PUUV-infected HUVEC, evaluated after 1, 2, 3, 4, 8 and 15 days of PUUV or mock infection. Results are shown as means of three independent technical replicates ± SD. Unpaired t-test was used to compare the percentage of PUUV-antigen positive and PUUV-antigen negative HUVEC between mock-infected and PUUV-infected HUVEC for each time point. **PUUV**: *Puumala orthohantavirus*.
(TIFF)

S7 Fig NK cell proliferation tested with CD56^+^ NK cells and peptide pulsed RMA-S/LFA3/HLA-E cells. AIPPAAYSL, VLPDSLFSF, LLPDKNLRM peptides were selected according to their %rank by NetMHCpan (http://www.cbs.dtu.dk/services/NetMHCpan/) and were identified by BLAST (SRKPRNFLL). The HLA-G leader peptide (VMAPRTLFL) served as a positive control. NK cells stimulated alone or with only RMA-S/LFA3/HLA-E cells served as a negative control. One representative example of three independent technical replicates is shown.
(TIFF)
