# Supplementary figures and images for: Dissection of the NKG2C NK cell response against Puumala Orthohantavirus
Source: PLoS Negl Trop Dis. 2021 Dec 6;15(12):e0010006. doi: 10.1371/journal.pntd.0010006 (PMC8714190; doi:10.1371/journal.pntd.0010006)

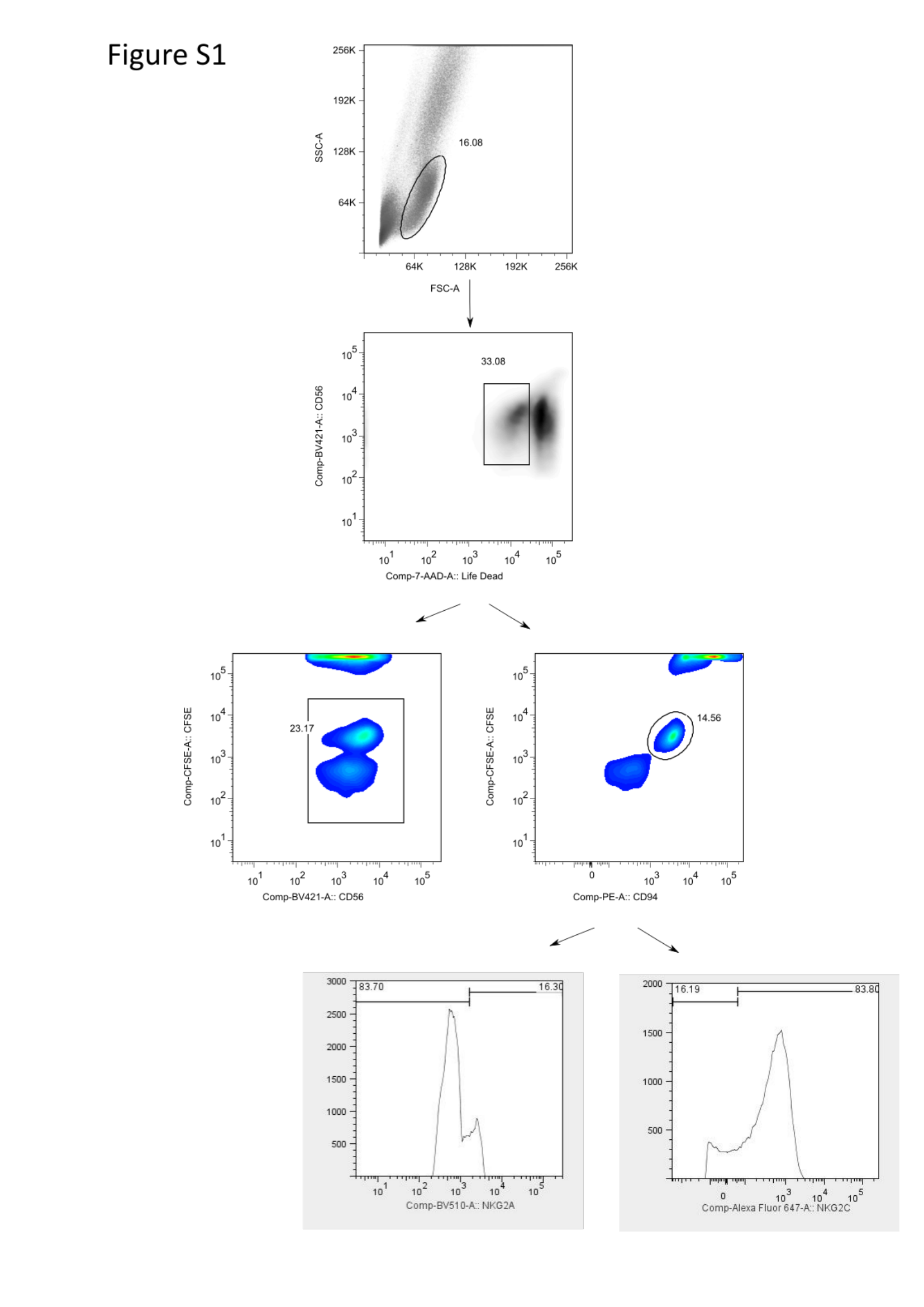

Supplement: S1 Fig — One representative example of 18 independent replicates is shown. (TIFF) [file pntd.0010006.s001.tiff]

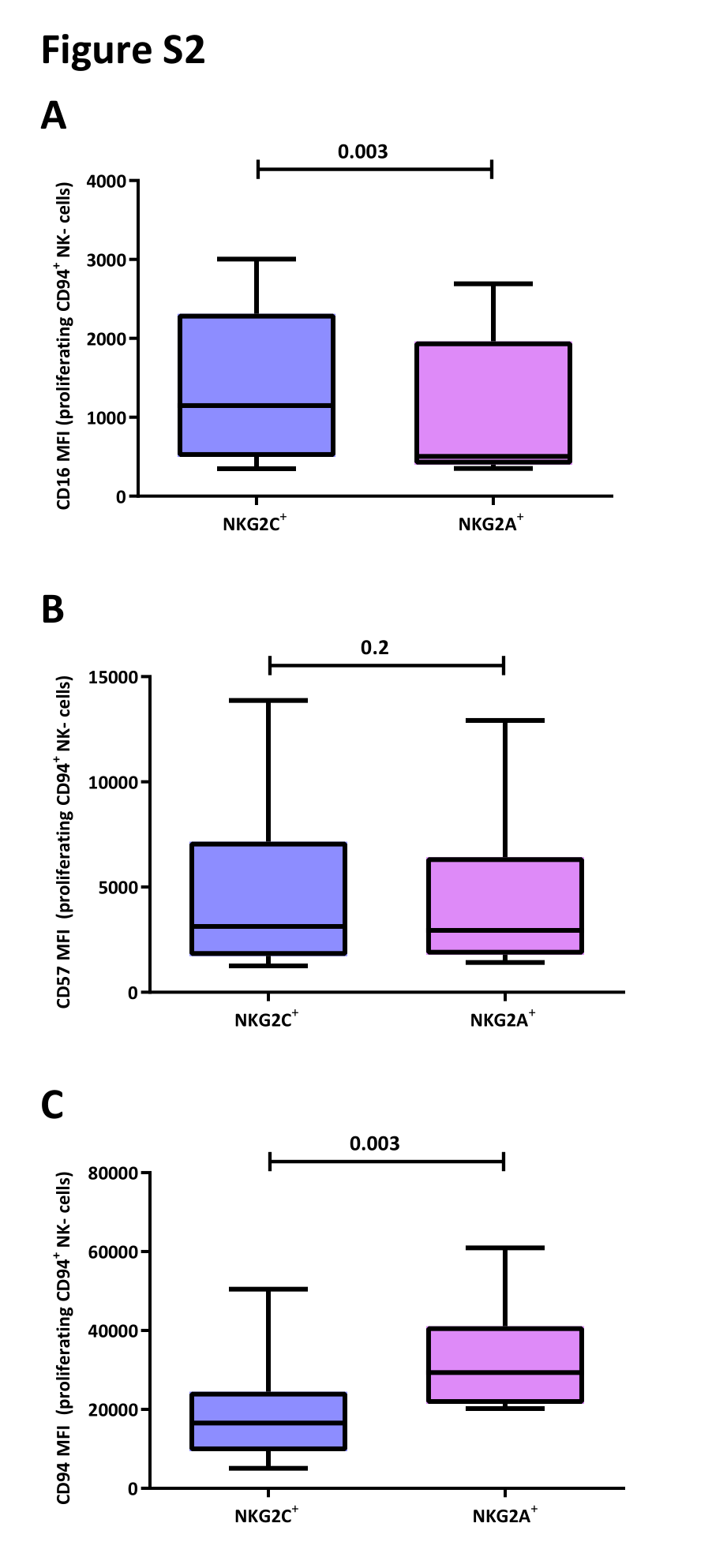

Supplement: S2 Fig — Expression level of (A) CD16, (B) CD57, and (C) CD94 measured in proliferating NKG2C+ and NKG2A+ NK cells obtained from 18 different healthy PUUV-naive human donors in response to PUUV-infected HUVEC in the presence of IL-15. The data of the 18 samples are shown as median ± min/max. Paired T-Test was used for statistical comparison. MFI: mean fluorescence intensity (TIFF) [file pntd.0010006.s002.tiff]

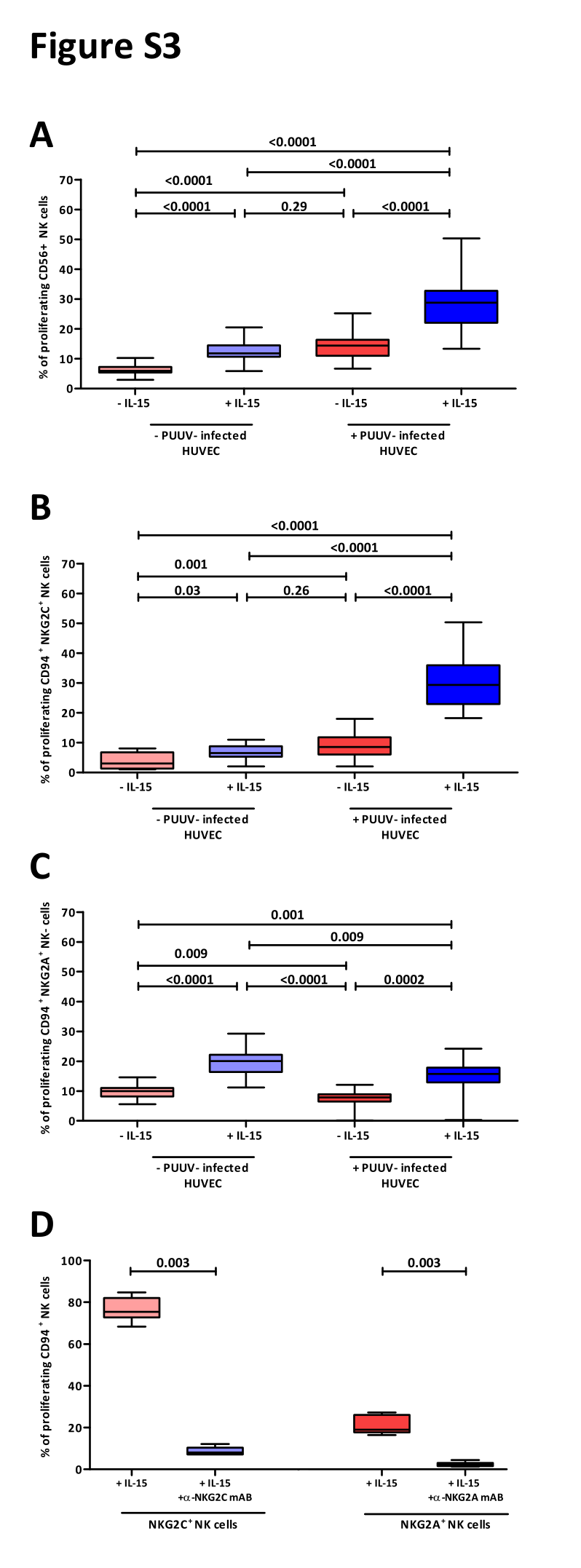

Supplement: S3 Fig — (A-C) Proliferation of (A) NKG2C+ cells, (B) NKG2A+ cells or (C) CD56+ NK cells obtained from 18 different healthy PUUV-naive human donors measured in response to NK cells cultured either without (-PUUV-infected HUVEC) or with PUUV-infected HUVEC (+PUUV-infected HUVEC) in the presence or absence of IL-15. The median ± min/max of the results of the 18 samples is shown. ANOVA and Dunn’s post test were used to compare the percentage of NK cells between mock-infected and PUUV-infected HUVEC. (D) Proliferation of NKG2C+ and NKG2A+ NK cells obtained from 6 different healthy PUUV-naive human donors (3 HCMV seropositive, 3 HCMV seronegative) measured in response to NK cells in the presence of IL-15 and the presence or absence of either a α-NKG2C or α-NKG2A blocking antibody. The median ± min/max of the results of the 6 samples is shown. Paired T-Test was used to compare the percentage of proliferating NK cells between the presence or absence of respective blocking antibodies. mAB: monoclonal antibody, PUUV: Puumala-Virus. (TIFF) [file pntd.0010006.s003.tiff]

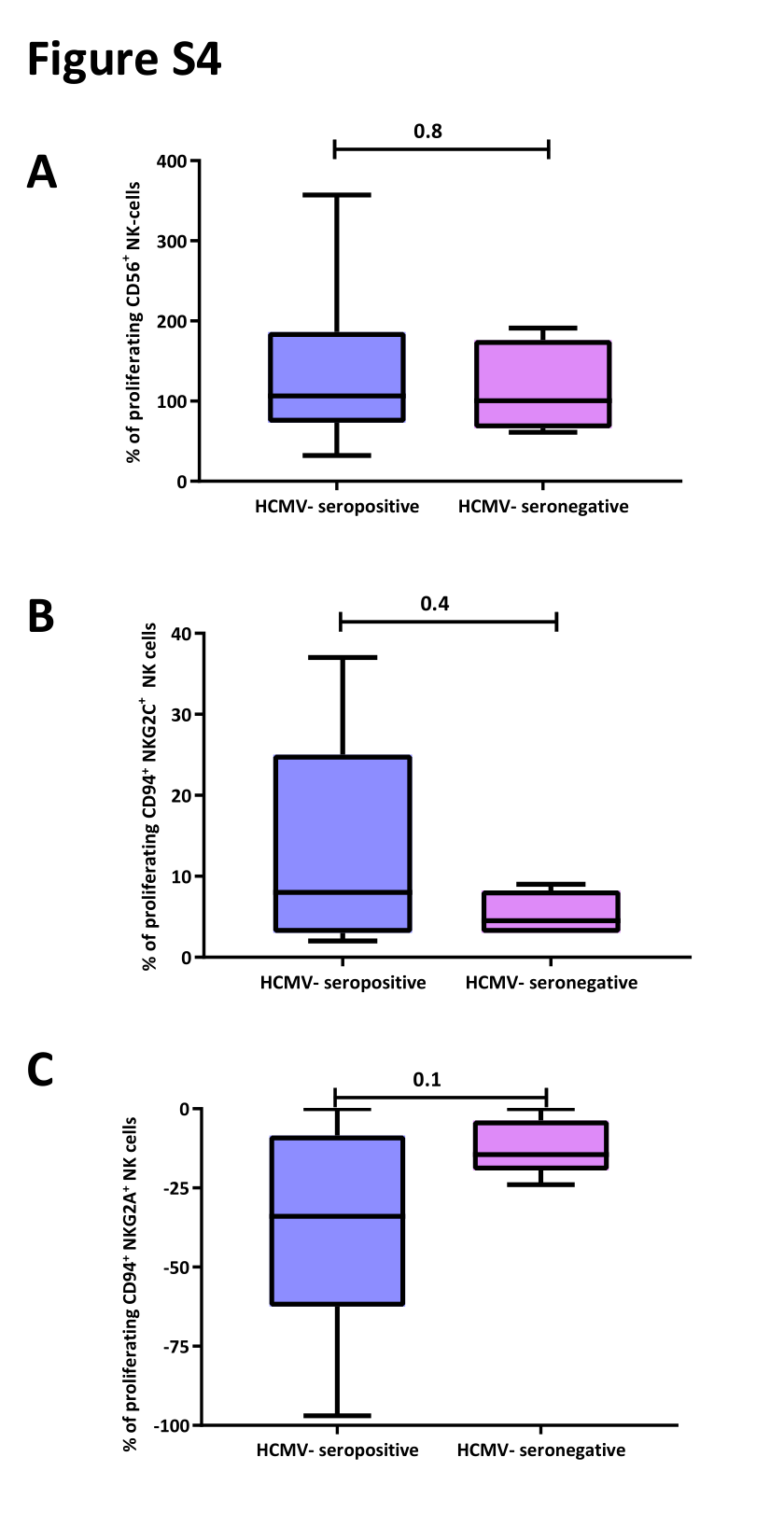

Supplement: S4 Fig — NK cell proliferation was measured in samples obtained from 18 different healthy PUUV-naive human donors, including HCMV-seropositive (N = 10) and HCMV-seronegative (N = 8) donors. Comparison of NK cell proliferation of (A) CD56+ (B) NKG2C+ and (C) NKG2A+ NK cells between HCMV-seropositive (N = 10) and HCMV-seronegative (N = 8) donors in the presence of IL-15. Data are shown as median of 18 different donors ± min/max. Mann-Whitney t-test was used to for statistical comparison between HCMV-seropositive and HCMV-seronegative donors. HCMV: Human Cytomegalovirus. (TIFF) [file pntd.0010006.s004.tiff]

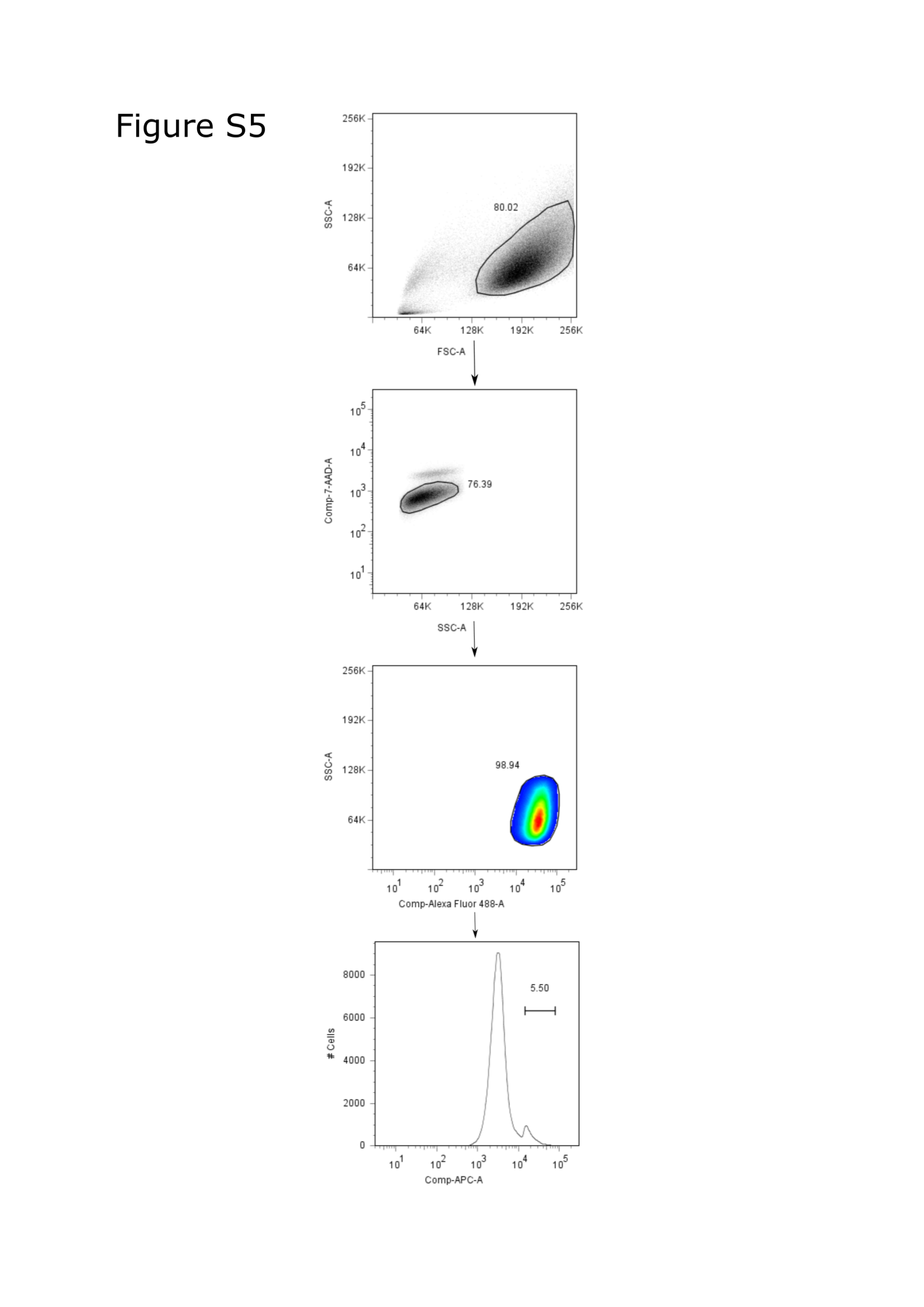

Supplement: S5 Fig — One representative example of 3 independent replicates is shown. (TIFF) [file pntd.0010006.s005.tiff]

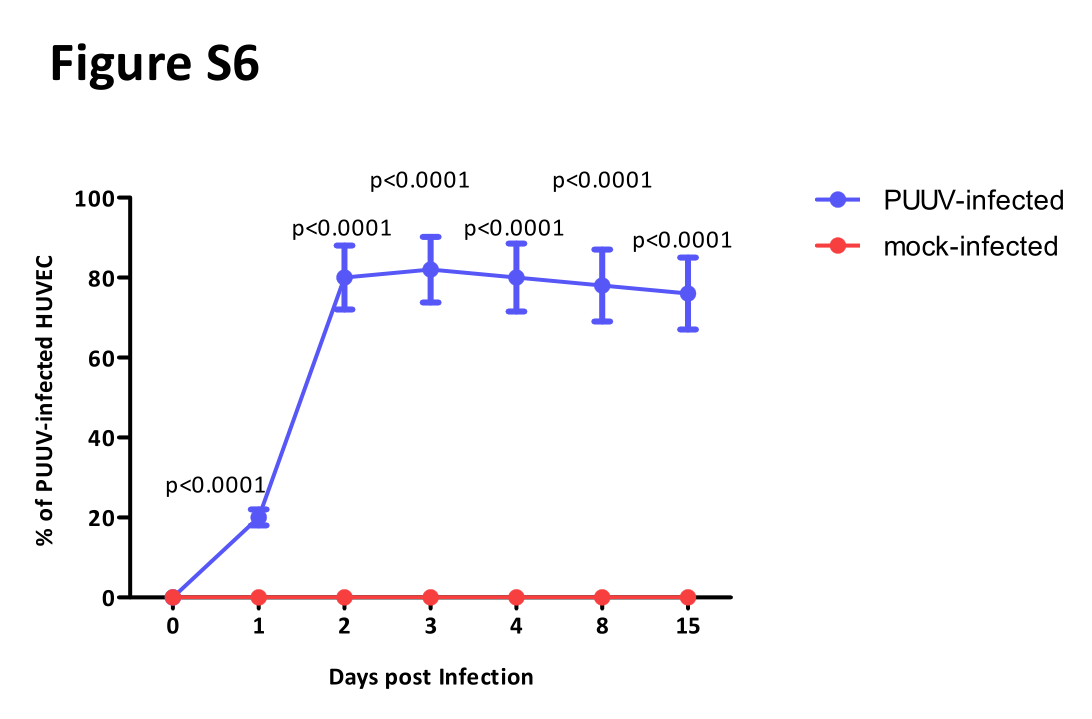

Supplement: S6 Fig — Kinetics of the PUUV-infection of HUVEC measured using flow cytometry. Percentage of PUUV-infected HUVEC, evaluated after 1, 2, 3, 4, 8 and 15 days of PUUV or mock infection. Results are shown as means of three independent technical replicates ± SD. Unpaired t-test was used to compare the percentage of PUUV-antigen positive and PUUV-antigen negative HUVEC between mock-infected and PUUV-infected HUVEC for each time point. PUUV: Puumala orthohantavirus. (TIFF) [file pntd.0010006.s006.tiff]

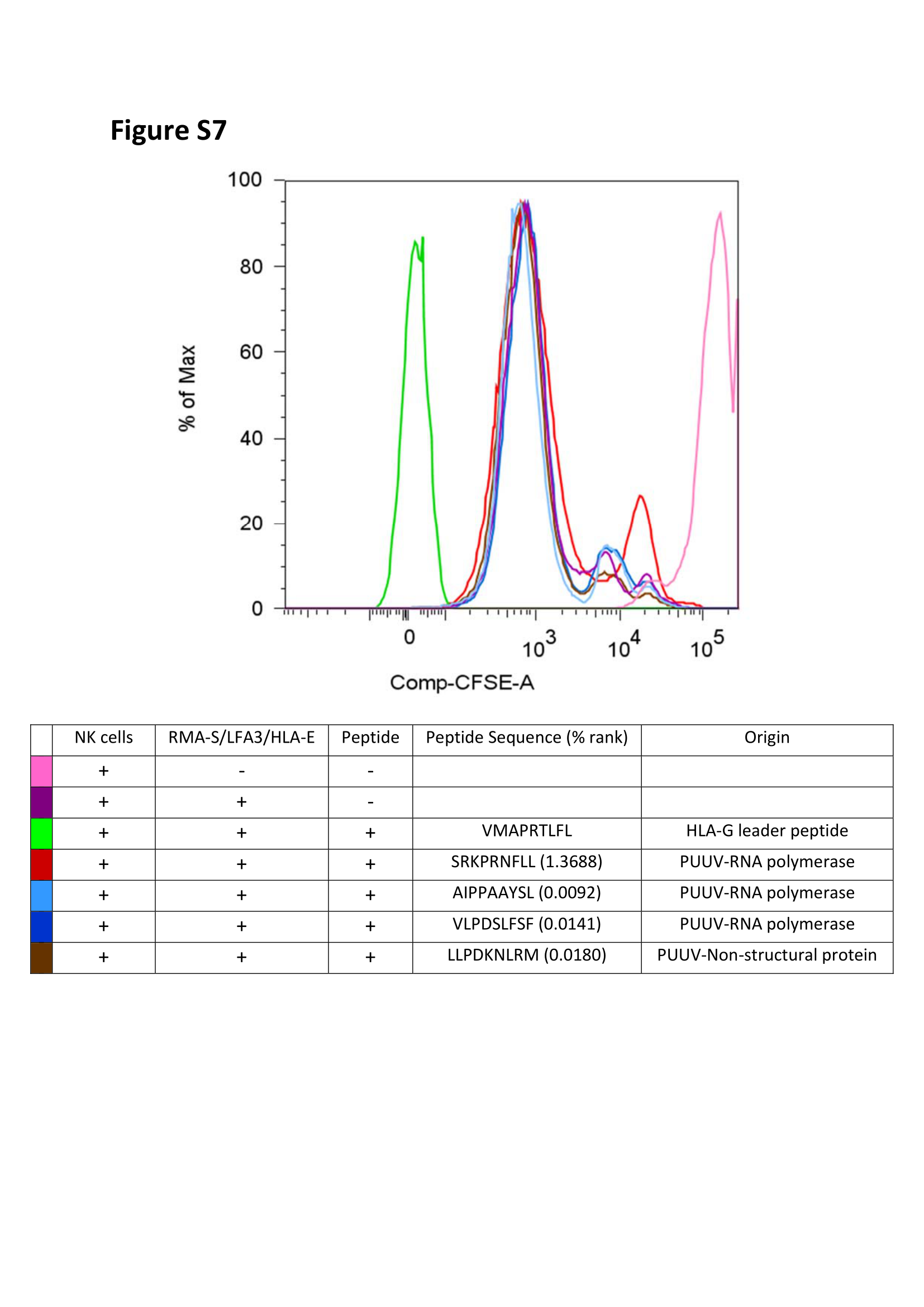

Supplement: S7 Fig — AIPPAAYSL, VLPDSLFSF, LLPDKNLRM peptides were selected according to their %rank by NetMHCpan (http://www.cbs.dtu.dk/services/NetMHCpan/) and were identified by BLAST (SRKPRNFLL). The HLA-G leader peptide (VMAPRTLFL) served as a positive control. NK cells stimulated alone or with only RMA-S/LFA3/HLA-E cells served as a negative control. One representative example of three independent technical replicates is shown. (TIFF) [file pntd.0010006.s007.tiff]
